# A Menace Worse Than War

**Published:** 1917-06

**Authors:** 


### A Menace Worse Than War.

The TEXAS MEDICAL JOURNAL has been shocked at the reports that have come to it about the rapid and alarming increase of syphilitic diseases among the soldiers of this country, and especially among those who have been on the border during the past year. So prevalent have syphilis and other venereal diseases become that the very strength and sinew of our army are imperiled as well as the future citizenship of the country. Something must be done, and must be done quickly to check it.

In the first place, radical steps must be taken to stop the horde of women camp followers who are demoralizing the army and ruining its health for their personal gain. These women in vast numbers are said to prey upon the young soldiers and tempt them to their moral and physical ruin. The country cannot have a virile soldiery if it permits them to become debauched and diseased, nor can it have a healthy nation with diseased fathers. The best blood of the country is going into its armies, and that blood must be saved from impurity. It is said that not only do these women carry their nefarious business into the camp neighborhoods but that in all the cities where soldiers visit they are accosted almost continually while on the streets, their uniforms making them easy marks for street walkers. The government should promptly penitentiary women for this offense.

In addition to heavy penalties for the women, the men, who are not wholly blameless, should also receive military punishment for such offenses as subject them to these disease. Then, they should be impressed in every way possible with the great danger to them as soldiers from contracting venereal diseases. Every encouragement should be given, together with financial assistance, to the movement now being made to offset this great army evil. It is not alone in the army, but in civil life the disease is said to to be spreading alarmingly. In some way vice seems to spread over a country in waves, and degeneracy flows in currents. While it is a delicate matter to discuss the evil of prostitution, it is, nevertheless, a thing that cannot be avoided if the evil is to be stopped. Doctors everywhere are in position, by reason of their profession, to use a powerful influence toward stopping the spread of the disease. They should not merely accept, they should seek opportunity to write and speak on the subject. Our young men and our young women as well should be impressed as to its lurking dangers, and the older people need to be informed as to its prevalence. Physicians need only to be reminded of a duty to do it, and the TEXAS MEDICAL JOURNAL is certain that there will be an early response to the many appeals being made for greater publicity on this subject.

The JOURNAL is arranging for a series of helpful articles on syphilitic diseases from physicians who know how to handle the subject with force and for the general good of the profession. The prominence to be given to the subject is justified by its grave importance.

